# Development of a questionnaire (EORTC module) to measure quality of life in patients with cholangiocarcinoma and gallbladder cancer, the EORTC QLQ-BIL21

**DOI:** 10.1038/sj.bjc.6606086

**Published:** 2011-01-25

**Authors:** E Friend, G Yadegarfar, C Byrne, C D Johnson, O Sezer, S Pucciarelli, S P Pereira, W-C Chie, A Banfield, J K Ramage

**Affiliations:** 1 Department of Gastroenterology and Hepatology, BNHFT Hospital Basingstoke, Aldermaston Road, Basingstoke RG24 9NA, UK; 2 Isfahan University of Medical Sciences, Isfahan, Iran; 3 University Hospital Aintree, Liverpool, UK; 4 Southampton University Hospitals, Southampton, UK; 5 Department of Oncology and Haematology, University Hospital, Berlin, Germany; 6 University of Padova, Padua, Italy; 7 University College London, London, UK; 8 National Taiwan University of Taiwan, Taipei, Taiwan; 9 Kings College Hospital, London, UK

**Keywords:** cholangiocarcinoma, gall bladder cancer, quality of life

## Abstract

**Background:**

Quality of life measurement in cholangiocarcinoma and gallbladder cancer involves the assessment of patient-reported issues related to the symptoms, disease and treatment of these tumours. This study describes the development of the disease-specific quality of life (QoL) questionnaire for patients with cholangiocarcinoma and gallbladder cancer to supplement the European Organization for Research and Treatment of Cancer (EORTC)-QLQ C30 core cancer questionnaire.

**Methods:**

Phases 1–3 of the guidelines for module development published by the EORTC were followed, with adaptations for incorporation of questions from existing modules.

**Results:**

A total of 47 QoL issues (questions) were identified; 44 questions from the two related validated questionnaires, the EORTC QLQ-PAN26 (pancreatic module) and the EORTC QLQ-LMC21 (liver metastases module), two from the Functional Assessment of Cancer Therapy hepatobiliary module questionnaire in the literature search and one from healthcare professional interviews. Following phase 1 and 2 interviews with patients (*n*=101) and health care professionals (*n*=6), a 23-question provisional questionnaire was formulated. There were five questions from PAN26, 15 from LMC21 and three extra questions. In phase 3, the provisional item list was pre-tested in 52 patients in four languages and this resulted in a 21-item module.

**Conclusion:**

This is the only disease-specific QoL questionnaire for patients with cholangiocarcinoma and gallbladder cancer, and initial assessments show it to be accurate and acceptable to patients in reflecting QoL in these diseases.

Quality of life (QoL) measurements are increasingly being used as endpoints in clinical trials especially those in patients with malignant diseases (Blazeby *et al*, 2002). A limited amount of research has been published about QoL in patients with cholangiocarcinoma and gallbladder cancer, and to our knowledge there is no disease-specific QoL score questionnaire.

Current cancer-specific QoL questionnaires are the European Organization for Research and Treatment of Cancer (EORTC) QLQ-C30 and the Functional Assessment of Cancer Therapy (FACT) generic questionnaires. These are supplemented by modules specific to different types of cancer because individual cancers have different etiologies, symptoms, treatments and prognosis (Blazeby *et al*, 2002). A FACT module for hepatobiliary cancers (FACT-Hep) is non-specific as it is designed for use in patients with cancer of the head of pancreas, colorectal hepatic metastases, primary liver cancer and cholangiocarcinoma. The EORTC QLQ-C30 does not address the specific symptoms of cholangiocarcinoma and gallbladder cancer, so there is a need to develop a disease-specific QoL questionnaire to supplement this.

There are other generic tools to measure QoL but they do not cover the specific issues that affect this group of patients. The pancreatic module, the EORTC QLQ-PAN26 (Fitzsimmons *et al*, 1999), which has issues related to tumours solely in the pancreas and the liver metastases module, the EORTC QLQ-LMC21 (Kavadas *et al*, 2003), which has issues related to liver metastases. However, these modules separately do not address the issues perceived by patients with cholangiocarcinoma and gallbladder cancer.

Cancers of the biliary tree include cholangiocarcinoma and gallbladder cancer, and are uncommon cancers, although there is evidence that the incidence of these has increased significantly over the last decade (McGlynn *et al*, 2006). In the United States, the incidences of cholangiocarcinoma and gall bladder cancer are 1–2 (Khan *et al*, 2005) and 2.5 cases (Kaushik, 2001) per 100 000 people per year, respectively. Cholangiocarcinoma incidence rates vary worldwide (Khan *et al*, 2008). The highest rates are in northeast Thailand (96 per 100 000 men) (Shaib and El-Serag, 2004). These cancers lie anatomically within the liver, and between the liver and pancreas, so issues relevant to the patients may be contained in the existing EORTC modules for pancreatic cancer (PAN26) (Fitzsimmons *et al*, 1999) and liver metastases (LMC21) (Kavadas *et al*, 2003).

Cholangiocarcinoma and cancers of the bile duct historically have been associated with poor prognosis and poor health-related quality of life (Heffernan *et al*, 2002). Cholangiocarcinoma presents a formidable challenge. The majority of patients present with unresectable disease and have a survival of less than 12 months (Anderson *et al*, 2004). Colorectal liver metastases patients have a median survival of 8 months without treatment (Garden *et al*, 2006), which is longer than patients with pancreatic cancer (4–8 months) (Yeo and Cameron, 1998) or extra-hepatic (6 months) and intra-hepatic (4 months) cholangiocarcinoma (Ahmed *et al*, 2008), implying some different issues for these tumours.

Clinical features of cholangiocarcinoma depend on the location of the tumour (Anderson *et al*, 2004). Symptoms are mainly related to intra- or extrahepatic biliary obstruction (DeOliveira *et al*, 2007; Weber *et al*, 2007). Intrahepatic cholangiocarcinoma can present with a mass in the liver but can obstruct local ducts causing fever and abscess formation. Extrahepatic cholangiocarcinomas cause obstruction with pruritus, jaundice and cholangitis. Gallbladder cancer can present with pain because of a mass and can also cause obstruction of the bile ducts by direct invasion. Systemic symptoms of cancer are common. The symptoms are similar to many of those present in pancreatic cancer, for example, anorexia, jaundice and itching but also may have similarities with symptoms of liver metastases such as right upper quadrant pain, local abscess formation with recurrent fevers and some patients may experience issues relating to external drains.

Treatments may be similar to both pancreatic and metastatic liver cancer—such as surgery (DeOliveira *et al*, 2007), stenting, external drainage (Patel and Singh, 2007), chemotherapy (Hong *et al*, 2007) and radiotherapy (Ben David *et al*, 2006). It has been difficult to show significant survival benefits of many treatments (Malhi and Gores, 2006; Hong *et al*, 2007), although a recent survival difference in two chemotherapy regimes has been reported (Valle *et al*, 2010) and those that do prolong life may actually reduce quality of life. A number of new therapies for cholangiocarcinoma are in clinical trial stage (for example, photodynamic therapy and new chemotherapy drugs) and it is important to have a validated QoL questionnaire to provide reliable data for a patient reported endpoint in these trials.

This paper describes the development of a specific module to accompany the EORTC QLQ-C30 to assess QoL in patients with cholangiocarcinoma and gallbladder cancer.

## Patients and methods

### Design

Guidelines for development of new modules published by the EORTC QoL group (Blazeby *et al*, 2002) describe three phases of module development: generation of issues from literature review, interviews with patients and interviews with health care professionals; conversion of these issues into a standard format list of items (provisional questionnaire) and initial testing in a different group of patients. These guidelines are designed for use where there are no existing modules for related cancers. Cholangiocarcinoma and gallbladder cancer are closely related to tumours of the pancreas and to liver metastases. We therefore modified phase 1 by relying heavily on the questions from the two existing modules for pancreas and liver metastases. Semi-structured interviews were performed with the list of questions and the core questionnaire QLQ-C30 as per the guidelines.

### Phase 1

#### Identification of issues

The QLQ-PAN26 and QLQ-LM21 were used to generate a list of issues. This was supplemented by issues raised from initial patient, and health care professional (HCP) interviews the literature search and a review of questions from FACT-Hep. Semi-structured interviews were performed with the list of questions and the core questionnaire QLQ-C30 as per the EORTC guidelines, in order to assess which questions were important, and if there were extra issues that had not been included.

The procedure followed is summarised in Table 1.

#### Subjects

Patients for the interviews for phase 1 were recruited from the UK, Taiwan and Germany.

The inclusion criteria were:

- Age 18 years and above.
- Diagnosis of cholangiocarcinoma or gallbladder cancer from histology or MDT (multidisciplinary team meeting/tumour board) agreement based on radiology or both.
- Patients able to give written informed consent
- No history of other significant malignant disease.
- Patients able to understand the language of the questionnaire.

Healthcare professionals were identified who had cared for patients with cholangiocarcinoma and gallbladder cancer.

Ethical committee permission was obtained.

#### Data collection

In phase 1, 101 patients and 6 HCPs were asked to rate the questions according to their relevance for quality of life for this group of patients on a four-point Likert scale (one—not at all, two—a little, three—quite a bit to four—very much) and to choose Yes/No if the question should be included in the questionnaire. They were asked if they had any other comments. HCPs were asked to list five questions they would like included in the questionnaire. The questions were grouped into clinically meaningful groups so the patients could compare items more easily, for example, the questions on eating were seen grouped together so they could be compared and chosen for relevance.

#### Data analysis

Descriptive statistics were used to analyse the results of the interviews in phase 1.

Semi-structured interviews were performed (at differing times before, during or after treatment) with the list of questions and the core questionnaire QLQ-C30. Patients were asked to respond to the questionnaire on how they have felt in the past week based on their total experience as result of illness and treatments.

### Phase 2: Selection of items

The questions were prioritised for inclusion based on an arbitrary scoring system. Table 2 lists the four categories where each carries a score of 0 or 1 and these were added to give a total score. Questions with total scores of 3 or more were provisionally included. Then the final decision was made after analysing comments made by the patients and HCPs.

### Phase 3: Pretesting

Patients were recruited from UK, Taiwan, Germany and Italy. The inclusion criteria were as for phase 1.

Recruitment was designed to interview at least 50 patients with the modified questionnaire and to have a reasonable spread of patients with intrahepatic, extrahepatic cholangiocarcinoma and gallbladder disease. It was also planned to have three treatment groups with 10–15 patients recruited per group. The treatment groups were surgery, chemotherapy/radiotherapy and supportive care including stent insertion/external drains.

In Phase 3, the Likert scores were assessed according to level of symptoms in 52 patients.

The ratings for relevance by mean Likert scores were linearly transformed to a 0–100 scale, with ‘not at all’ corresponding to 0 and ‘very much’ to 100 (for example, pretransformed score 2=33.3 on ‘transformed’ score) (Garden *et al*, 2006). Items with mean scores of less than 25 were considered for deletion, but retained if clinically appropriate and either patients or professionals considered them sufficiently important to test further. Ranges were examined to ensure a reasonable spread of scores.

Patients were asked to complete six debriefing questions and were asked if any of the questions were difficult, annoying, confusing, upsetting, intrusive or irrelevant. They were asked if there were any questions that they thought were relevant but not included in the provisional questionnaire and if they had any other comments.

Construct validity was assessed by discriminant validity and convergent validity by Pearson's correlation. To explore convergent validity correlation between items and scales was performed. For investigating discriminant validity, correlation between single items and other scales were calculated. Criterion validity refers to how well one variable or set of variables predicts an outcome based on information from other variables. This can be achieved by known group comparisons and these were curative *vs* noncurative intent and performance status.

## Results

### Phases 1 and 2

#### Literature search

Literature searches were performed in five databases (Medline, Embase, Cinhal, AMED and Psych Info) to locate relevant articles. Quality of life was linked with the following number of articles: cholangiocarcinoma (43), gallbladder cancer (0) and pancreatic cancer (28). A manual search of relevant issues within these papers was done, as well as a search within the alternative questionnaires. From that, a provisional list of 47 questions and issues was made.

#### Interviews with HCPs

Six HCPs (one gastroenterologist, one hepatologist, one registrar, two nurse specialists and one nursing sister from hospice) were interviewed.

There were 18 questions that HCPs selected for inclusion in the module. Health care professionals rated each question's relevance and wanted nine questions to be included. These were taken into account in the scoring system (Table 2).

#### Interviews with patients

Clinical details of the patients are shown in Table 3.

There was a representative spread of patients with intrahepatic *vs* extrahepatic disease as well as a reasonable number of patients with gallbladder cancers. Patients were recruited into three treatment groups (at least 10–15 per group) and this was in similar proportions as that seen in clinical practice. Some patients had more than one treatment so the total percentage for all treatments was more than 100%. Patients were interviewed (many recently diagnosed) between 1–72 months from diagnosis with mean of 8.9 and median of 3.5 months.

#### Analysis, adaptation and production of the provisional questionnaire

A total of 47 QoL issues were generated from the related validated questionnaires PAN26 and LMC21, literature search and HCP interviews. A total of 44 existing items were used, as three replicates were excluded. Existing questions were used except for the question on jaundice. The new question ‘Have you been worried about your skin being yellow?’ was generated from HCP interviews, but an equivalent question from the EORTC QoL item bank could not be found. There were three extra issues that were included, the question about jaundice and two questions from the FACT-hep questionnaire ‘Did you have fevers?’ and ‘Did you have any difficulties with drainage tubes/bags?’

Questions/issues were deleted on the basis of the scoring system after taking into account comments made by patients’ and HCPs, to form the provisional item list to be used in Phase 3.

Questions were deleted: one each from eating and jaundice scales, five from the relationship scale, two from the pain scale, three from the body image scale, both questions from the bowel scale, two from the satisfaction scale and six single items.

The question on fevers was kept in because of scoring and was highlighted as important by HCPs and patients. The question on tubes and bags was included as HCPs thought it was clinically relevant.

This led to the number of items being reduced from 47 to 23. Five questions from PAN26, 15 from LMC21 and 3 extra questions formed a provisional list of 23 items.

The items were grouped into five scales of eating, jaundice, tiredness, pain and anxiety and five additional items, which did not fit into the scales.

The questions were translated according to QoL Group translation guidelines into Chinese Mandarin, Italian and German (Dewolf *et al*, 2009).

#### Phase 3: pretesting the provisional item list

Pretesting interviews were performed using the provisional questionnaire of 23 questions together with the QLQ-C30 on patients (*n*=52) from the UK, Taiwan, Germany and Italy.

Linear transformation of Likert scores are listed below, in Table 4, with items that scored <25 highlighted in red. Debriefing questions were completed by 52 patients and 15 comments were made. These included support for questions on jaundice and tubes/bags, but no specific comments relating to possible deletion of questions. Other comments made by more than one patient in these interviews were that jaundice had a big impact on QoL, and that family and emotional issues were considered important.

All questions had ranges from 1 to 4 and had no obvious ‘floor’ or ‘ceiling’ effects, which would necessitate deletion.

On the basis of interviews and descriptive statistics, two questions were deleted. These were single item questions F1 (had fevers) and L46 (trouble talking about feelings to family or friends). Question F2 (difficulties with drainage tubes/bags) was kept in the questionnaire even though the ‘transformed’ mean score was<25 because clinical opinion suggested this was an important question for the relatively few patients who have tubes or bags. Question X in the jaundice scale (worried about skin being yellow) was kept in, even though the ‘transformed’ mean score was <25. This was because Cronbach's alpha for the jaundice scale (with question X removed) decreased to an unacceptable figure of 0.58 from an acceptable figure of 0.78 (with question X included). This may be because this question is applicable for only some patients, but for those patients suffering from jaundice the question was very important. The new questionnaire has 21 items and is called the EORTC QLQ-BIL21. It has five multi-item scales: eating (four items); jaundice (three items); tiredness (three items); pain (four items) and anxiety (four items) and three single items about treatment side effects, drainage tubes/bags and worried about losing weight.

Although the number of patients interviewed was limited, psychometric analysis was performed with following results.

### Construct validity

This was assessed by Pearson's correlation.

For convergent validity, all the named scales and related questions had significant correlations of *r*⩾0.40, which shows the questions in the named scales are measuring the same construct and working well together.

For discriminant validity all correlations were less than 0.40. This shows items were not highly correlated with other scales as expected as different scales are measuring different groups of issues. Correlations between scales were also less than 0.40, which confirmed the scales were measuring defined groups of issues.

There was no strong correlation between scales in the core module and new module.

### Criterion validity

This was assessed by known group comparisons:

1. Patients treated with curative intent (surgery) were compared those with noncurative intent (stent insertion, chemotherapy, radiotherapy). Treatment with curative intent was associated with significantly lower mean scores for jaundice (12 *vs* 32), pain (19 *vs* 37), single item – worried about losing weight (9 *vs* 29) and anxiety (borderline at 41 *vs* 56) with noncurative intent.
2. Performance status was correlated to each scale and was significantly related to the scales eating (mean score for Karnofsky <70, 46 *vs* 22 for ⩾70), jaundice (mean score for Karnofsky <70, 35 *vs* 15 for ⩾70), tiredness (mean score for Karnofsky <70, 78 *vs* 50 for ⩾70) and two single items (difficulties with drainage tubes and treatment side effects) (mean score for Karnofsky <70, 50 *vs* 20 for ⩾70).

### Internal consistency

This was assessed using Cronbach's alpha coefficient, which was >0.70 for the proposed scales indicating good internal consistency (Table 5).

## Discussion

The EORTC QLQ-BIL21 (in Table 6) is a disease-specific module for patients with cholangiocarcinoma and gallbladder cancer to be used in addition to the core questionnaire EORTC QLQ-C30. Together, these questionnaires assess all aspects of health-related QoL in these patients. The QLQ-BIL21 assesses issues related to disease, treatment and emotional wellbeing, which are not covered by the QLQ-C30. It has been developed using adapted EORTC module development guidelines, see Table 1.

As the clinical symptoms, treatment and prognosis of cholangiocarcinoma are closely related to those of pancreatic cancer and liver metastases, it was possible to develop this module by combination of relevant items from existing modules for pancreas cancer (PAN26) and liver metastases (LMC21). In addition, issues raised from initial patient and HCP interviews, and the literature search were included.

The QLQ-BIL21 has five questions from PAN26, 14 from LMC21 and 2 extra questions (one from FACT-Hep and one from HCP interviews). The QLQ-BIL21 has been pretested in four languages (three in Europe, one in Asia). Our study has shown that the QLQ-BIL21 is relevant, acceptable and applicable to patients in different countries and cultures. The questionnaire appears in our preliminary testing to have high construct and convergent validity as well as good reliability and internal consistency. All these features will require confirmation in a Phase 4 field test.

Generally, cholangiocarcinoma and gallbladder cancer are not detected until late in the disease course, thus significant symptoms and QoL issues rapidly develop. This is reflected in the issues captured in the questionnaire. Patients score very highly on the tiredness and the anxiety scales, which seem to affect the majority of patients. As cholangiocarcinomas can be small but can cause obstruction of bile ducts, the issues around jaundice are particularly relevant. Gallbladder cancers are often larger and may have a mass effect, leading to pain and jaundice. The systemic symptoms of cancer were common in this group of patients and in particular those described in the eating scale. The question on difficulty with drainage tubes did not score highly but was very important to patients who experienced this, and clinical opinion reinforced this. Bile-duct cancer in particular often requires treatment with percutaneous drainage tubes, which reinforces the need for a specific module for this tumour type. The question on side effects of treatment scored highly with patients, thus, both quality and quantity of life are important factors when deciding on treatments, hence, assessment of QoL is needed to justify the use of these therapies.

There were some limitations of the study. Cholangiocarcinoma and gallbladder cancer are less common diseases so it was difficult to recruit large numbers of patients in a reasonable timescale. In all, 100 patients were recruited in phase 1 and 2 (as with modification to the guidelines, it was felt appropriate to include a large number of patients at this stage) and 50 patients in phase 3. These numbers are comparable with many other published phase 3 studies of module development and are consistent with the guidelines for module development (Valle *et al*, 2010). The psychometric evaluations would normally be done on a larger cohort of patients but the results imply good results for this sample. The patients in phase 3 were described as receiving treatment in three separated but overlapping groups: (1) surgery, (2) chemotherapy and radiotherapy and (3) supportive care, including external drains and stent insertion. Many patients received more than one treatment as is shown in Table 3 and the distribution of these treatments in phase 3 patients is comparable with that seen in clinical practice, suggesting our sample is appropriate for development of a clinically relevant module. This preliminary analysis will be confirmed later in the phase 4 study with a larger international sample. More European than Asian patients were included for phases 1–3, but the higher incidence in Asia will necessitate higher percentage recruitment from Asia in phase 4. Another limitation is that, although a number of HCPs from different fields were selected for interview for phase 1, these did not include oncologist, radiologist or surgeon.

The EORTC QLQ-BIL21 questionnaire will now undergo international field-testing to confirm the reliability, validity and cross-cultural applicability.

## Conclusion

This questionnaire that is specific for use in patients with cholangiocarcinoma and gallbladder cancer will supplement the QLQ-C30 and should detect small changes in QoL because of disease progression or treatments.

## Figures and Tables

**Table 1:** Process of development for cholangiocarcinoma and gallbladder module

**Phase**	**Aims**	**Process**
1	Generation of QoL issues relevant to the selected group of patients.	1. Use all questions from PAN26 and LMC21 2. Literature search 3. Interviews with health care professionals 4. Patients’ interviews 5. Analysis of quantitative and qualitative data and use of scoring system to adapt list of issues
2	Construction of a provisional questionnaire	1. Consultation of EORTC QoL group item database 2. Construction of new items 3. Translation of provisional questionnaire
3	Pretesting to identify and solve potential problems	1. Interview including administration of questionnaire to patients 2. Quantitative and qualitative data analysis 3. Modification of questionnaire 4. Formal development report reviewed by EORTC QoL group

Abbreviations: EORTC=European Organization for Research and Treatment of Cancer; QoL=quality of life.

**Table 2:** Scoring system for selection of items in phase 1

**Scoring criteria for questions**	**Threshold**	**Score**
Mean of patients’ Likert score	>1.5	1 (0 for <1.5)
<20% patients said no to inclusion	>20%	1 (0 for>20%)
<30% HCP said no to inclusion	>30%	1 (0 for >30%)
HCP said include in top 5		1

Abbreviation: HCP=health care professional.

**Table 3:** Demographic and clinical details of patients in Phases 1–3

**Demographics**	**Phase 1 and 2, *n*=101**		**Phase 3, *n*=52**	
Country of recruitment (%)	Germany	21 (21)	Germany	5 (10)
	Taiwan	4 (4)	Italy	3 (6)
	UK	76 (75)	Taiwan	10 (19)
			UK	34 (65)
Age (years)	Mean (s.d.)	64 (12)	Mean (s.d.)	67 (8.3)
	Median	67	Median	67
	Range	28–87	Range	50–80
Male (%)		51 (50.4)		25 (48)
Site (%)	Intrahepatic	36 (36)	Intrahepatic	24 (46)
	Proximal extrahepatic^a^	31 (31)	Proximal extrahepatic^a^	14 (27)
	Distal extrahepatic^a^	13 (12)	Distal extrahepatic^a^	5 (10)
	Gallbladder	21 (21)	Gallbladder	8 (15)
	Unknown	0 (0)	Unknown	1 (2)
Treatment (%)	1. Surgery	42 (42)	1. Surgery	22 (42)
	2. Chemotherapy and/or radiotherapy (including PDT)	44 (44)	2. Chemotherapy and/or radiotherapy	25 (48)
	3. Supportive care includes: stent insertion	54 (54)	3. Supportive care includes external drain/stent insertion	51 (98)
Karnofsky status before treatment	Mean (s.d.)	74 (16)	Mean (s.d.)	73 (15.4)
	Median	80	Median	80
	Range	20–100	Range	40–100

a Proximal extrahepatic=above cystic duct and distal extrahepatic=below cystic duct.

**Table 4:** Linear transformation of mean Likert scores for each question/item

**Question**	**Proposed scale**	***N***	**Mean**	**Standard deviation**
L31-trouble eating	Eating	52	27.6	30.0
L32-fullness after eating	Eating	51	37.9	30.6
L34-taste problems	Eating	51	24.8	31.2
P36-restricted in food intake	Eating	51	30.7	30.4
L41-skin/eyes yellow	Jaundice	51	26.1	38.5
P44-had itching	Jaundice	51	23.5	32.9
*X-worried skin yellow*	*Jaundice*	*50*	*17.3*	*30.3*
L37-less active	Tired	51	60.1	32.0
L43-slowed down	Tired	51	58.2	30.0
L44-lacking in energy	Tired	51	62.1	29.9
P34-pain at night	Pain	51	30.1	32.1
L39-pain in stomach	Pain	51	28.1	30.8
L42-pain in back	Pain	51	26.8	32.7
P32-bloating in abdomen	Pain	51	35.9	33.2
L47-felt stressed	Anxiety	51	34.6	29.0
L48-less able to enjoy yourself	Anxiety	50	57.3	33.7
L49-worried about future health	Anxiety	51	54.9	32.5
L50-worried about family in future	Anxiety	51	54.2	38.9
P50-treatment side effects	Single item	51	39.9	35.3
*F1-had fevers*	*Single item*	*51*	*17.0*	*28.6*
*F2-difficulties with drainage bags/tubes*	*Single item*	*49*	*17.7*	*30.5*
*L46-trouble talking about feelings to family/friends*	*Single item*	*51*	*19.0*	*26.9*
L33-worried about losing weight	Single item	51	27.5	31.8

Those in italics were considered for deletion—see text.

**Table 5:** Internal consistency—Cronbach's alpha values

	**Cronbach's alpha**
Eating	0.76
Jaundice	0.78
Tiredness	0.94
Pain	0.83
Anxiety	0.80

**Table 6:** EORTC QLQ-BIL21

**During the past week**	**Not at all**	**A little**	**Quite a bit**	**Very much**
31. Have you had trouble with eating?	1	2	3	4
32. Have you felt full up too quickly after beginning to eat?	1	2	3	4
33. Have you had problems with your sense of taste?	1	2	3	4
34. Were you restricted in the types of food you can eat as a result of your disease or treatment?	1	2	3	4
35. Have your skin or eyes been yellow (jaundiced)?	1	2	3	4
36. Have you had itching?	1	2	3	4
37. Have you been worried about your skin being yellow	1	2	3	4
38. Have you been less active than you would like to be?	1	2	3	4
39. Have you felt ‘slowed down’?	1	2	3	4
40. Have you felt lacking in energy?	1	2	3	4
41. Did you have pain during the night?	1	2	3	4
42. Have you had pain in your stomach area?	1	2	3	4
43. Have you had pain in your back?	1	2	3	4
44. Did you have a bloated feeling in your abdomen?	1	2	3	4
45. Have you felt stressed?	1	2	3	4
46. Have you felt less able to enjoy yourself?	1	2	3	4
47. Have you worried about your health in the future?	1	2	3	4
48. Were you worried about your family in the future?	1	2	3	4
49. To what extent have you been troubled with side-effects from your treatment?	1	2	3	4
50. Have you had difficulties with drainage tubes/bags?	1	2	3	4
51. Have you worried about losing weight?	1	2	3	4
